# Factors associated with a reduced food intake after third molar extraction among adults: a cross-sectional study

**DOI:** 10.1186/s12903-022-02154-w

**Published:** 2022-04-11

**Authors:** Shinichi Taniguchi, Atsushi Abe, Yu Ito, Takanori Ishihama, Hiroki Hayashi, Moeko Momokita, Ryuta Naito, Kanae Shibata

**Affiliations:** Department of Oral and Maxillofacial Surgery, Nagoya Ekisaikai Hospital, Nagoya, 454-8502 Japan

**Keywords:** Tooth extraction, Third molar, Food intake

## Abstract

**Background:**

Functional impairment after third molar extraction may decrease the food intake. Elucidation of associated factors will contribute to a more appropriate postoperative nutritional management, and was the aim of the present study.

**Methods:**

Adults aged < 60 years who were admitted for an extraction of one or more mandibular third molars were included. Those with diabetes mellitus, anemia, metabolic diseases, mental retardation, altered dietary intake, and postoperative paralysis of the lower lip and tongue were excluded. Patient-specific risk factors were compared in relation to a decrease in the food intake on postoperative day 1. Multivariate analysis took into account the patients’ background factors.

**Results:**

A total of 254 patients were included (median age: 26.8 ± 9.3 years, 142 women); 508 third molars were extracted. Postoperative dietary intake reduction was more common (*p* < 0.05) after an exclusively mandibular extraction (16.0%) than after an extraction including the maxilla (29.4%). The reduction was also more common (*p* < 0.05) for an extraction difficulty of Pell–Gregory class III (39.5%) than for extraction difficulties of Pell–Gregory classes I (22.6%) and II (21.3%). The reduction was also more common (*p* < 0.05) in patients who experienced postoperative pain (66.7%) than in those who did not (23.3%). Significant differences were observed in sex (women: 34.5%, men: 11.6%) and age (young patients [< 26 years]: 31.1%, adult patients [≥ 26 years]: 17.2%); however, no significant difference was found in terms of experiencing trismus (*p* < 0.11). Simple regression analysis showed significant differences between patients who did and did not have a reduced postoperative food intake depending on the sex, age, extraction site, degree of extraction difficulty, trismus, and postoperative pain. Reduced dietary intake was significantly associated with sex (odds ratio [OR]: 0.30; 95% confidence interval [CI]: 0.14–0.38), age (OR: 1.6; 95% CI: 1.0–2.5), extraction site (OR: 0.51; 95% CI: 0.31–0.83), difficulty of extraction (OR: 0.66; 95% CI: 0.50–0.88), and postoperative pain (OR: 0.12; 95% CI: 0.04–0.37).

**Conclusions:**

A younger age, female sex, extraction including the maxilla with deep implantation, and complaints of pain on postoperative day 1 were factors associated with a decreased food intake after third molar extraction.

## Background

Oral health is essential for maintaining the general health and well-being at all stages of life. The oral environment, which included the oral microbiota and their biofilms, can cause a variety of infections and affect the nutritional status [1–3]. Discomforts, such as pain and trismus, occur in approximately 7% of the patients after a third molar extraction, particularly after a mandibular third molar extraction [4, 5]. These discomforts affect chewing and swallowing functions and may reduce the postoperative food intake; this is of great concern in these patients. Several factors, including biomarkers [6], are involved in decreased postoperative food intake. For instance, chemotherapy causes oral mucositis. Oral mucositis causes pain, gingival swelling, and ulceration. Therefore, oral mucositis can be considered as a cause of decreased food intake. However, there are no reports on the extent of discomfort that affects food intake after tooth extraction.

Adequate nutritional intake is necessary for wound healing. The reference energy intake for adults is 1950–2800 kcal/day [7]. Therefore, if difficulties in postoperative dietary intake can be predicted, the management of postoperative nutrition can be improved. There are various reports on tooth extraction; however, most detail studies on techniques and complications. Similarly, there are many studies on nutritional management; however, most were conducted among patients with cancer. No studies have been performed on the nutritional management of patients after a third molar extraction.

Thus, the main purpose of this retrospective study was to investigate the factors involved in the reduction of food intake after a third molar extraction and to clarify the statistical differences. We hypothesized that trismus, postoperative pain, difficulty of extraction, and extraction site would affect the amount of post-extraction food intake. It is anticipated that our findings will contribute to a more appropriate postoperative nutritional management by clarifying the actual situation of decreased food intake after a third molar extraction.

## Methods

### Patients

Patients who were admitted to the Department of Oral and Maxillofacial Surgery (Nagoya Ekisaikai Hospital) for a third molar extraction between April 2018 and May 2021, who met the inclusion criteria, and who provided written informed consent for the purpose and content of this study were included.

The inclusion criteria were as follows: (1) extraction of at least one mandibular third molar, (2) age below 60 years (age on the date of providing consent), and (3) healthy status or a history of disease (provided the exclusion criteria were not met). Due to their influence on the food intake, the exclusion criteria were as follows: (1) pre-existing diabetes mellitus, anemia, metabolic diseases, mental retardation, and other conditions requiring modification of food intake and (2) postoperative paralysis of the lower lip and tongue.

The present study was approved by the Ethics Committee of the Nagoya Ekisaikai Hospital (No. 2021-017). All patients and volunteers were informed of the aims of the study and provided their written consent before participation. The study was conducted in accordance with the Strengthening the Reporting of Observational Studies in Epidemiology guidelines.

### Procedures

#### Preoperative management

The patient was admitted to the hospital at 10:00 a.m. on the day of the surgery (postoperative day 0) after fasting from 8:00 a.m. After securing the intravenous route at 2:00 p.m., an antibacterial agent (flomoxef sodium) was administered; the extraction procedure was performed under intravenous sedation (15 mg pentazocine, 10 mg diazepam). Sedation was performed in accordance with the Practice Guidelines for Intravenous Conscious Sedation in Dentistry (Second Edition, 2017), which were developed by the Japanese Dental Society of Anesthesiology for Dentists Practicing Sedation in Japan [8].

#### Surgical procedure

All surgeries were performed by a single dentist; affiliated to the Japanese Society of Oral and Maxillofacial Surgeons, they are a specialist in oral and maxillofacial surgery and have more than 20 years of clinical experience.

After disinfecting the oral cavity, surface anesthesia was applied to the local anesthetic administration site. Using a 27-gauge needle (Disposable Needle, Terumo Corporation, Tokyo, Japan), the mandibular foramen was anesthetized with one Xylocaine® cartridge (1.8 mL) containing epinephrine. Local anesthesia (1.8 mL; ORA Injection Dental Cartridge 1.8 mL, SHOWA YAKUHIN KAKO CO., LTD. Tokyo, Japan) was infiltrated around the mandibular third molar to the buccal gingiva using a 31-gauge needle. Using a no. 15 scalpel (Surgical Blade, FEATHER Safety Razor Co., Ltd, Tokyo, Japan), the dentist then made a gingival incision, thereby creating a three-cornered flap [9]. The mucoperiosteal flap was dissected and a sufficiently wide surgical field was secured. A turbine (OF1-SP, Osada, Inc. Tokyo, Japan) was used for bone removal. The crown was divided using a round bar; it was removed subsequently. Finally, the tooth root was removed using a hevel (Dental Elevators #3, #5. YOSHIDA DENTAL TRADE DISTRIBUTION CO., LTD. Tokyo, Japan). The wound was closed and no drainage was used [10].

#### Postoperative management

**Fig. 1:**
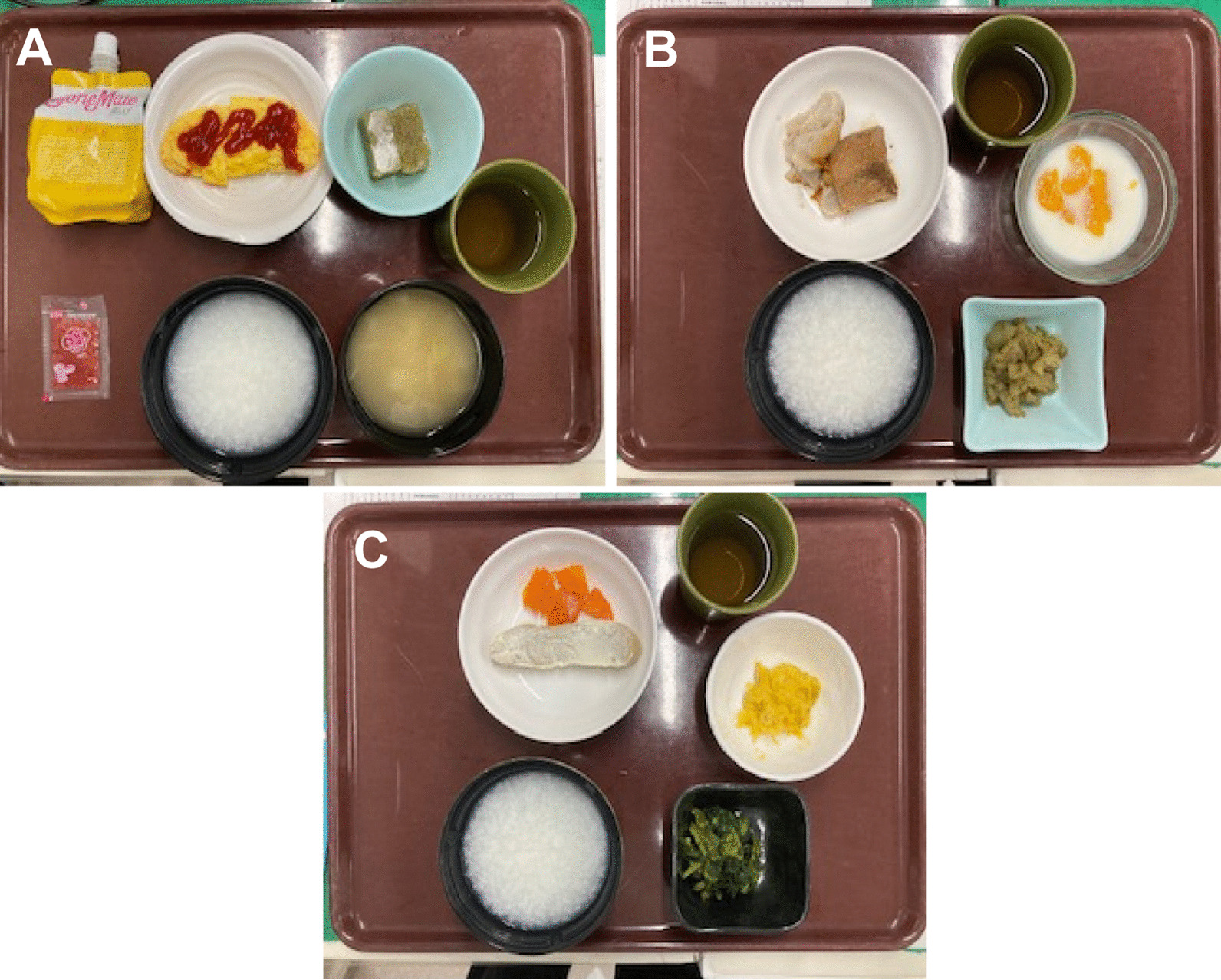
An example of the meals served at our hospital: **A** breakfast, **B** lunch, and **C** dinner

After returning to their hospital room, an analgesic (loxoprofen sodium) was prescribed, and the patients could consume it when required. However, if the Numerical Rating Scale (NRS) score was 4 or higher, the patients were instructed to take the analgesic. The patients were administered with an intravenous antimicrobial infusion 6 h after surgery, although no other prescribed medication was provided. On the day of the surgery, dinner was served at 6:00 p.m.; however, on postoperative day 1, the patients received three meals, starting in the morning (Fig. 1). They were discharged after breakfast on postoperative day 2.

**Table:** 

The content and structure of each plate						
	Energy (Kcal)	Protein (g)	Lipids (g)	Carb (g)	Salt (g)	Structure
(A)	540	20	11	90	2	Soft solid
(B)	430	15	11	60	1	Soft solid
(C)	430	15	13	60	2	Soft solid
Total	1400	50	35	210	5	-

The outcome of this study was the amount of food intake on postoperative day 1, given that our clinical process for third molar extraction involves the extraction procedure being performed in the afternoon on the day of the surgery (postoperative day 0) and dinner being served immediately thereafter. Moreover, on the day of the discharge (postoperative day 2), only breakfast is served. Therefore, the daily intake could only be measured on postoperative day 1. Patients who deviated from the clinical process were excluded from the study. Reasons for deviation included early discharge for personal reasons, additional administration of antimicrobial agents due to postoperative infection, and prolonged hospitalization.

#### Dietary intake assessment

Two trained ward nurses recorded the patient data in the medical records under the direction of the physician. Data on the dietary intake, assessed on a 10-point scale (0 = no intake at all; 10 = maximum intake), were obtained from these records. The total energy content of breakfast, lunch, and dinner was 1400 kcal (Fig. 1). The preoperative food intake could not be measured because the patients were admitted on the day of the surgery after being *nil per os* since the morning; hence, we were unable to compare the preoperative and postoperative intake. Consequently, referring to the Japanese Ministry of Health, Labour and Welfare's Manual for Improving Nutrition [11], the reduced dietary intake group included those patients who consumed 88 kcal or less—which is less than 20% of the maximum intake—in at least one meal among the three meals on postoperative day 1. Dietary intake at dinner on the day of the surgery was not considered in the analysis because the patients were still affected by the sedative drugs administered during the surgery.

### Statistical analyses

The selected predictor variables were classified as follows, after which the decrease in dietary intake on postoperative day 1 was compared between the groups: (1) sex (male or female); (2) age at surgery (< 26 years [young] or ≥ 26 years [adult]); (3) extraction site (including the maxilla or exclusively the mandibular); (4) difficulty of extraction (Pell–Gregory classification classes I/II/III); (5) trismus on postoperative day 1 (yes or no); and (6) pain on postoperative day 1 (NRS score < 7 [painless] or ≥ 7 [pain]). In our study, preoperative panoramic radiographs were used to evaluate the degree of expected surgical difficulty. Because the difficulty of extraction is related to the depth of implantation [12], we examined the depth of implantation, which was classified into Pell–Gregory classes I–III [13].

**Fig. 2:**
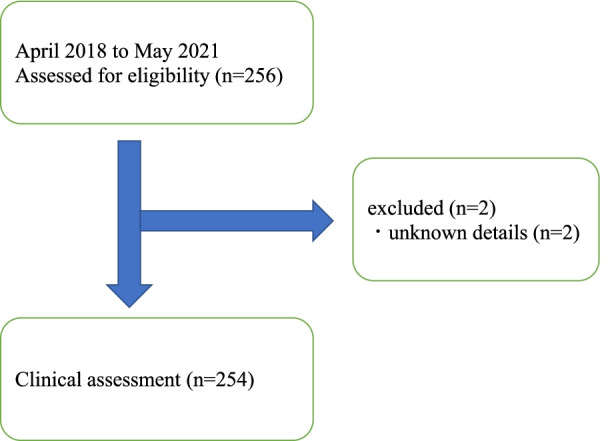
Flowchart of the study

The R package, EZR [14] (Saitama Medical Center, Jichi Medical University, Saitama, Japan), was used for all statistical analyses. *t*-tests were conducted for group comparisons when the Kolmogorov–Smirnov test demonstrated normality of the data. For data that were not normally distributed, the χ^2^ test and the Fisher’s exact test were used. Multivariate analysis was performed because correlation was observed for all items in the simple regression analysis. Logistic regression analysis was performed employing a model in which the outcome comprised the presence or absence of a reduction in the food intake, and the variables were difficulty of extraction, postoperative pain, and other covariates. Odds ratios (ORs) and 95% confidence intervals (CIs) were calculated. The covariates were sex, age, extraction site, and trismus on postoperative day 1. The statistical significance level was set at 5%. Statistical analysis was performed by a trained third party who was not familiar with the study. The required sample size was calculated at a 5% significance level. The total number of potentially eligible participants was 256. However, 254 participants were included after excluding two patients for whom the required data were not available (Fig. 2).

## Results

The data of 254 patients (median age: 26.8 ± 9.3 years, 142 women), who had 508 third molars extracted, were analyzed (Table 1). Among them, 62 (24.4%) experienced a reduction in the dietary intake; 2 (0.79%) were unable to consume any of the three meals, 16 (6.3%) were unable to consume two meals, and 44 (17.3%) were unable to consume only one meal.

**Table 1:** Preoperative patient characteristics

Items		Number	%
Total number of patients		254	100
GenderSex	Female	142	55.9
	Male	112	44.1
Age (years)			
Median (range)	26.8 ± 9.3 (19–59)	254	100
Site	Full	118	46.5
	Bilateral mandibular	88	34.6
	Left mandibular	5	2.0
	Right mandibular	1	0.40
	Bilateral mandibular + either maxilla	31	12.2
	Other	11	4.3
Difficulty	Class I	239	47
(Pell-–Gregory Classification)	Class II	188	37
	Class III	81	16
Trismus	Yes	57	22.4
	No	197	77.6
Postoperative pain	0	140	27.6
(NRS score)	1	58	11.4
	2	172	33.8
	3	68	13.4
	4	28	5.5
	5	14	2.8
	6	10	2.0
	7	12	2.4
	8	4	0.80
	9	0	0
	10	2	0.40

*NRS* numerical rating scale

Comparisons were drawn within the groups related to the extraction site, difficulty of extraction, trismus, and pain. A postoperative reduction in the dietary intake was significantly more common in patients who underwent an extraction that included the maxilla as compared to in those who underwent an exclusively mandibular extraction (mandibular: 15/94, 16.0%; including the maxilla: 47/160, 29.4%). The postoperative reduction in the dietary intake was also significantly more common in patients with a Pell–Gregory class III difficulty of extraction (class I: 54/239, 22.6%; class II: 40/188, 21.3%; class III: 32/81, 39.5%) and in those who experienced postoperative pain (pain: 12/18, 66.7%; painless: 114/490, 23.3%). Furthermore, significantly more women (49/142, 34.5%) than men (13/112, 11.6%) and more young patients (41/132, 31.1%) than adults (21/122, 17.2%) had a reduced postoperative dietary intake. Conversely, the number of patients with a postoperative reduction in the dietary intake did not differ significantly between those who experienced trismus and those who did not (with: 9/57, 15.8%; without: 53/197, 26.9%; *p* < 0.11). Simple regression analysis revealed significant differences between patients who did and did not experience a reduction in the dietary intake depending on the sex, age, extraction site, difficulty of extraction, trismus, and postoperative pain (*p* < 0.05; Table 2).

**Table 2:** Preoperative outcomes

	Number with eating	Number with non-eating	*p* value
Sex (n = 254)			*p* < 0.05
Female (n = 142)	93	49	
Male (n = 112)	99	13	
Age (n = 254)			*p* < 0.05
Young (n = 132)	91	41	
Adult (n = 122)	101	21	
Site (n = 254)		X	*p* < 0.05
Only mandibular (n = 94)	79	15	
Including maxilla (n = 160)	113	47	
Difficulty (n = 508)			
(Pell–Gregory Classification)			
Class I (n = 239)	185	54	*p* < 0.05
Class II (n = 188)	148	40	
Class III (n = 81)	49	32	
Trismus (n = 254)			*p* = 0.11
Yes (n = 57)	48	9	
No (n = 197)	144	53	
Postoperative pain (n = 508)			*p* < 0.05
Painless (n = 490)	376	114	
Pain (n = 18)	6	12	

χ^2^, Fisher’s exact tests

Logistic regression analysis was subsequently performed; multicollinearity was ignored based on a calculated variance inflation factor of below 10. A reduced dietary intake was significantly associated with sex (OR: 0.30; 95% CI: 0.14–0.38), age (OR: 1.6; 95% CI: 1.0–2.5), extraction site (OR: 0.51; 95% CI: 0.31–0.83), difficulty of extraction (OR: 0.66; 95% CI: 0.50–0.88), and postoperative pain (OR: 0.12; 95% CI: 0.04–0.37) (Table 3). It was found that young patients, women, patients who underwent extraction that involved a high degree of difficulty, patients who exclusively underwent mandibular extraction, and patients who complained of pain on postoperative day 1 experienced a significant reduction in the food intake.

**Table 3:** Multivariate analysis

Factor	Odds ratio	95% CI	*p* value
Sex	0.30	0.14–0.38	*p* < 0.05
Age	1.6	1.0–2.5	*p* < 0.05
Site	0.51	0.31–0.83	*p* < 0.05
Difficulty	0.66	0.50–0.88	*p* < 0.05
Postoperative pain	0.12	0.04–0.37	*p* < 0.05

Logistic regression analysis

*CI* confidence interval

## Discussion

We found that the factors associated with a decreased food intake in patients after a third molar extraction included a younger age, female sex, extraction involving the maxilla with deep implantation, and complaints of pain on postoperative day 1. In the future, studies with a larger sample size are needed to validate these findings. Biomarkers have been implicated in inflammation and nutrition [6]. The bacterial origin of inflammation begins with a biofilm-bacterial imbalance, which leads to the regulation of inflammatory cytokines and ultimately determines the sprouting of oxidative stress cells [15, 16]. Saliva is said to be the protective agent of the oral cavity [17], and systemic conditions can affect the oral health by decreasing the saliva volume and altering the balance of the oral microbiota.

This study explored the factors involved in the reduction of food intake after a third molar extraction. We found that a reduced dietary intake was significantly more common in patients who experienced postoperative pain, involved class III difficulty of extraction, and underwent an exclusively mandibular extraction. However, no significant difference in the postoperative intake was found among those who did and did not experience trismus. We also found a significant difference in the sex and age. Thus, except for the findings on trismus, our hypothesis was confirmed.

Trismus on postoperative day 1 did not affect the food intake. This may be because even with trismus, the patients were able to ingest foods with a fluid- or paste-like consistency. Thus, malnutrition could be avoided by manipulating the consistency of the meal.

Postoperative pain had a significant effect on the food intake. Capuzzi et al. [18] reported that the surgeon’s skill level significantly affects postoperative pain. Only one dentist performed the extractions in all the participants in this study, without variation in the technique or the skill. Given that pain is subjective, we used the NRS—an objective, validated pain assessment tool [19]—in this study. Postoperative pain is affected by the incision method, age of the patient, and difficulty of extraction [20]. Nageshwar [21], who conducted a randomized controlled trial, reported that the degree of pain is significantly lower with comma incisions than with conventional incisions. However, the significance of the comma incision is unclear, because the details of the conventional incision method were unspecified and the difficulty of the extraction was unclassified.

In the present study, the incision was made using a three-cornered flap [8]. The lidocaine/adrenaline bitartrate, flomoxef sodium, pentazocine, and diazepam used during the surgery were identical for all patients. Monaco et al. [22] reported no significant difference in pain between the group of patients who received postoperative antimicrobial administration and the group who did not. However, we used flomoxef sodium, which was not included in their study. For intravenous sedation, we used pentazocine and diazepam. Both drugs have side effects of postoperative nausea and vomiting. According to Hasegawa et al. [23], these side effects are considered as sufficiently resolved when patients are examined on the first day after surgery. Moreover, no significant differences in postoperative pain have been observed in terms of the placement or non-placement of sutures or the use of different drugs [24].

Assessing the difficulty of surgery preoperatively is effective for planning treatment that avoids postoperative functional impairment leading to challenges in food intake. The amount of food intake was expected to decrease as the difficulty of the third molar extraction increased. We found that the amount of food intake decreased significantly in patients with class III difficulty, suggesting that the degree of difficulty of third molar extraction is one of the factors affecting the amount of food intake.

In the present study, the extraction site was classified as exclusively mandibular and inclusive of the maxilla. In a study by Yuasa et al. [25], compared to patients who underwent an exclusively mandibular extraction, those who underwent an extraction involving the maxilla showed a significantly decreased postoperative dietary intake, but no increased postoperative pain. Damage to the lateral and medial pterygoid muscles affects trismus.

Compared to men, women had a significantly reduced postoperative dietary intake. This may be due to differences in the postoperative pain experienced by men and women. Benediktsdóttir [19] reported that compared to in men, postoperative pain was more severe in women at 1 week postoperatively. Conversely, Capuzzi et al. [20] reported that compared to women, men predominantly experienced more pain on the first and third days postoperatively. Thus, the results of the two studies are not in agreement. In our study, there was no multicollinearity between sex and pain (variance inflation factor < 10); hence, the dietary intake of men and women was considered as a factor separate to pain. It is said that women are more affected by mental health [26], which may be one of the factors.

At our institution, meals are disposed of 1 h after serving for hygiene reasons. Therefore, a consistent meal temperature was maintained in this study. Patients who had not consumed their meal by the time that it was disposed of were considered to have no intention of consuming it. The Nutrition Improvement Manual of the Japanese Ministry of Health, Labour and Welfare defines poor dietary intake as consuming less than 75% of the total reference intake [27]. Ravasco et al. [28] found that a determinant of the quality of life was nutritional intake in 20% of the patients, although this was specific to certain cancers. In our study, the amount of food consumed during hospitalization was recorded by a nurse on a scale of 10; the amount varied by approximately 10% depending on the subjectivity of the person measuring it. Therefore, we defined patients who consumed less than 20% of their total dietary intake as the reduced diet group.

We hypothesized that the difficulty of extraction would be a cause of postoperative dietary intake reduction; nonetheless, we found that it was a less significant cause than other factors. Age, however, was a significant factor. This may be due to surgical duration and postoperative swelling. Benediktsdóttir [29] categorized the study participants into adult and young patients, and compared their operative durations; they noted that the operative duration was longer in the adult patient group. The reason for this is unknown; however, it is believed that this may be because the periapical bone is softer at a younger age while the third molars tend to adhere to the periapical bone at an older age. The median age in our study was 26.8 ± 9.3 years, with the young group aged below 26 years and the adult group aged 26 years and above. Although the age of comparison was different between this study and the study by Benediktsdóttir, the findings suggest that there is no relationship between the surgical duration and the postoperative food intake.

In addition to the variables measured in the present study, there may be other variables that confound postoperative swelling. Yuasa and Sugiura [20] conducted a two-group comparison of age and postoperative swelling, and found that the adult group had significantly more swelling than the young group. However, in our study, there was a reduced dietary intake in the young group, suggesting that there is no relationship between postoperative swelling and a decreased food intake. There is no published evidence that suggests a direct association between age and postoperative swelling. Moreover, there is a lack of evidence verifying this association, and further research is needed. Therefore, our results may not be objective in this regard. We secured the sample size (N = 254) that was required for the statistical analyses. However, the multivariate analysis included few cases (n = 62), which reduced the statistical power. Further prospective studies are needed, with the above considerations taken into account.

This study had certain limitations. The amount of food consumed was measured by two trained nurses; therefore, there are individual differences and an inherent bias in the measurers. Thus, there is a possibility that the results may not be objective. We were able to measure the dietary intake only on the first postoperative day; measuring the dietary intake on more days would be preferential. Measurement of the intake during each meal and of each nutrient would enable better nutritional management. Recently, the introduction of the Enhanced Recovery After Surgery [30] protocol has received much attention; it suggests providing patients with a nutritional supplement 4 h after surgery and again on the first day after surgery in addition to meals. Patient-controlled feeding [31] is also worthy of attention; this protocol involves providing food to postoperative patients at the time that they would like to eat. It is not necessary that all patients eat the same meals; however, it is necessary to improve individual responses and ensure that the meals are appropriate for every individual. More use should be made of the Nutrition Support Team activities.

## Conclusions

We found that the factors associated with decreased food intake in patients after a third molar extraction included a younger age, female sex, extraction involving the maxilla with deep implantation, and complaints of pain on postoperative day 1. In future, studies with a larger sample size are needed to validate these findings.

## Data Availability

The raw data are confidential and cannot be shared readily. Researchers are required to obtain permission from the Institutional Review Board and apply for data access from The Ethics Committee of the Nagoya Ekisaikai Hospital.

## Abbreviations

CI: Confidence interval
NRS: Numerical rating scale
OR: Odds ratio
